# The Impact of Time Horizon on Classification Accuracy: Application of Machine Learning to Prediction of Incident Coronary Heart Disease

**DOI:** 10.2196/38040

**Published:** 2022-11-02

**Authors:** Steven Simon, Divneet Mandair, Abdel Albakri, Alison Fohner, Noah Simon, Leslie Lange, Mary Biggs, Kenneth Mukamal, Bruce Psaty, Michael Rosenberg

**Affiliations:** 1 Division of Cardiology University of Colorado School of Medicine Aurora, CO United States; 2 University of Colorado School of Medicine Aurora, CO United States; 3 Department of Epidemiology University of Washington Seattle, WA United States; 4 Department of Biostatistics University of Washington Seattle, WA United States; 5 Division of Biomedical Informatics and Personalized Medicine University of Colorado Anschutz Medical Campus Aurora, CO United States; 6 Cardiovascular Health Research Unit University of Washington Seattle, WA United States; 7 Department of Medicine Beth Israel Deaconess Medical Center Boston, MA United States; 8 Department of Medicine University of Washington Seattle, WA United States

**Keywords:** coronary heart disease, risk prediction, machine learning, heart, heart disease, clinical, risk, myocardial, gender

## Abstract

**Background:**

Many machine learning approaches are limited to classification of outcomes rather than longitudinal prediction. One strategy to use machine learning in clinical risk prediction is to classify outcomes over a given time horizon. However, it is not well-known how to identify the optimal time horizon for risk prediction.

**Objective:**

In this study, we aim to identify an optimal time horizon for classification of incident myocardial infarction (MI) using machine learning approaches looped over outcomes with increasing time horizons. Additionally, we sought to compare the performance of these models with the traditional Framingham Heart Study (FHS) coronary heart disease gender-specific Cox proportional hazards regression model.

**Methods:**

We analyzed data from a single clinic visit of 5201 participants of a cardiovascular health study. We examined 61 variables collected from this baseline exam, including demographic and biologic data, medical history, medications, serum biomarkers, electrocardiographic, and echocardiographic data. We compared several machine learning methods (eg, random forest, L1 regression, gradient boosted decision tree, support vector machine, and k-nearest neighbor) trained to predict incident MI that occurred within time horizons ranging from 500-10,000 days of follow-up. Models were compared on a 20% held-out testing set using area under the receiver operating characteristic curve (AUROC). Variable importance was performed for random forest and L1 regression models across time points. We compared results with the FHS coronary heart disease gender-specific Cox proportional hazards regression functions.

**Results:**

There were 4190 participants included in the analysis, with 2522 (60.2%) female participants and an average age of 72.6 years. Over 10,000 days of follow-up, there were 813 incident MI events. The machine learning models were most predictive over moderate follow-up time horizons (ie, 1500-2500 days). Overall, the L1 (Lasso) logistic regression demonstrated the strongest classification accuracy across all time horizons. This model was most predictive at 1500 days follow-up, with an AUROC of 0.71. The most influential variables differed by follow-up time and model, with gender being the most important feature for the L1 regression and weight for the random forest model across all time frames. Compared with the Framingham Cox function, the L1 and random forest models performed better across all time frames beyond 1500 days.

**Conclusions:**

In a population free of coronary heart disease, machine learning techniques can be used to predict incident MI at varying time horizons with reasonable accuracy, with the strongest prediction accuracy in moderate follow-up periods. Validation across additional populations is needed to confirm the validity of this approach in risk prediction.

## Introduction

Cardiovascular disease (CVD) is the leading cause of morbidity and mortality in the United States and worldwide. The prevalence of CVD in adults within the United States has reached 48% and greater than 130 million adults in the United States are projected to have CVD by 2035, with total costs expected to reach US $1.1 trillion [1]. The leading cause of deaths attributable to CVD are from coronary heart disease, followed by stroke, hypertension, and heart failure [1]. This year alone, roughly 605,000 Americans will have an incident myocardial infarction (MI) and greater than 110,000 will die from MI [1]. Given the high prevalence of MI, there is significant focus on identifying those most likely to develop incident coronary heart disease [2-5]. If properly identified, primary preventive pharmacologic and lifestyle strategies can be applied to those at the highest risk [6].

Historically, risk prediction models have been developed by applying traditional statistical models (ie, regression-based models and Cox) to cohort data [7-10]. These analyses have provided a breadth of information about the risk of CVD and have been very useful clinically, given their straightforward relationships between a small number of variables and the outcome of interest [11-16]. However, these risk scores often do not achieve high reliability when applied to novel data sets [10,17]. Currently, roughly half of MIs and strokes occur in people who are not predicted to be at an elevated risk for CVD [18].

Machine learning has been introduced as a novel method for processing large amounts of data, focused primarily on accurate prediction rather than understanding the relative effect of risk factors on disease. In some applications, machine learning methods have been found to improve upon traditional regression models for predicting various cardiovascular outcomes [19-22]. A key aspect of applying machine learning methods is the bias-variance trade-off or balancing how accurately a model fits the training data (bias) and how well it can be applied broadly (variance) in out-of-sample testing or validation data [23]. Machine learning models tend to excel when dealing with a large number of covariates and nonlinear or complex relationships of covariates, often at the expense of overfitting a particular training set [24]. However, with an increased ability to model complex interactions between covariables comes a decrease in understanding how risk factors relate to an outcome. Additionally, one key limitation of many machine learning methods is that they are often classification models that do not include well-developed methods to incorporate information about time-to-event data. Investigators often select a single time horizon for classification, but how varying time horizons affect the relative prediction accuracy is a relatively unexplored aspect of machine learning methods. We hypothesize that there is a trade-off in the selection of the predictive time horizon, in which the use of shorter time horizons offers an increased relevance of predictors to outcomes and greater effect sizes. This is balanced against an increase in the number of events when the time horizon is of longer duration. Based on this trade-off, we would predict that moderate time horizons would have the highest predictive accuracy.

With this investigation, we examined the impact of varying time horizons on the prediction of incident MI. Using data from the Cardiovascular Health Study (CHS) [25], we examined the predictive accuracy of multiple machine learning algorithms over varying time frames of 500 days through 10,000 days of follow-up to identify incident MI. Additionally, we used the Framingham Heart Study (FHS) coronary heart disease gender-specific Cox proportional hazards regression model for comparison to the machine learning models. We aimed to find what time horizon would have the highest predictive accuracy and examine how this compared with the prediction accuracy of the FHS regression model.

## Methods

### Ethical Considerations

Data were approved for use by the Cardiovascular Health Study Policies and Procedures Committee with accompanying data and materials distribution agreement.

### Data Set Creation

We used anonymized data from the CHS [25], the design and objectives of which have been previously described. Briefly, the CHS is a longitudinal study of men and women aged 65 years or older, recruited from a random sample of Medicare-eligible residents of Pittsburgh, PA, Forsyth County, NC, Sacramento, CA, and Hagerstown, MD. The original cohort of 5201 participants was enrolled in 1989-1990 and serves as the sample for this study. Baseline data were obtained in this cohort, and routine clinic visits and telephone interviews were conducted periodically going forward.

We excluded patients with a baseline history of prior MI from the cohort. We examined 61 variables collected from the baseline exam, including demographic and biologic data (Table S1 in Multimedia Appendix 1).

**Figure 1:**
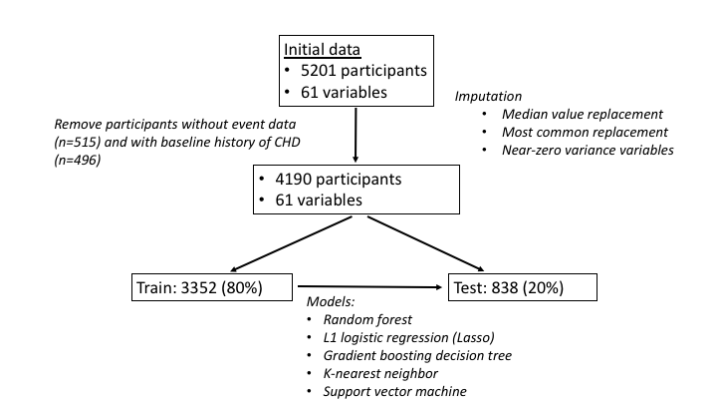
Analysis flowchart. CHD: Cardiovascular Health Study.

Using an end point of incident MI, we applied multiple machine learning methods across varying time horizons to define an optimal risk prediction. Missing variable data was quite uncommon for baseline demographic and laboratory data. Although overall infrequent, missing data was more common for electrocardiogram variables. In these cases of missing data, imputation was performed on missing variables using median value replacement for continuous variables and most common replacement for categorical variables (Figure 1).

### Statistical Analysis

The data set was randomly split into a training set (80%) and a testing or validation set (20%). The training data set was used to construct 5 machine learning models: random forest, L1 (LASSO) regression, support vector machine, k-nearest neighbor, and gradient boosted decision tree. Hyperparameter tuning to identify the optimal values for parameters that are not learned during the training process was performed using the validation set. These models were then applied to the test set to examine model performance, which was assessed using an area under the receiver operating characteristic curve (AUROC). Additionally, we used the FHS coronary heart disease Cox proportional hazards regression model as a comparison to the machine learning models (Table S2 in Multimedia Appendix 1) [7,9,26].

Starting at 500 days, we looped each model over 500-day time horizons in order to identify the optimal predictive horizon up through 10,000 days of follow-up time. For each time horizon, variable importance algorithms were applied to the L1 regression and random forest models. In the L1 regression model, coefficients that are less helpful to the model were shrunk to zero, thereby removing unneeded variables altogether. The remaining coefficients are the variables selected. Because models use normalized inputs, direct comparison of coefficients can be performed based on the absolute value of the average coefficient for each input. In the random forest algorithm, we performed a “permutation” feature selection, which measures the prediction strength of each variable by measuring the decrease in accuracy when a given variable is essentially voided within the model.

Preliminary analyses identified a high degree of bias related to the cases that were selected within the held-out split sample, and so we performed 50 analyses with different random seeds, with separate results stored for each model, time horizon, and seed number (a total of 1000 separate models for each type of model). Results were compiled based on the average AUROC, coefficient value (L1 regression), and impurity or accuracy (random forest) for each model. Model comparison was performed using linear mixed effects models, with seed number as the random effect and unstructured covariance matrix pattern.

All modeling was performed using publicly available packages on R software (version 1.1.463; The R Foundation for statistical computing). The code used for analysis is provided in Multimedia Appendix 1. Model comparisons (mixed effects models) were performed using Stata IC (version 14; Stata, Inc).

## Results

Baseline characteristics of the study participants are presented in Table 1. There were a total of 4190 participants included. The average age of the cohort was 72.6 years, and 2522 (60.2%) participants were female. At baseline, 2201 (53 %) had a history of ever using tobacco, 2300 (55%) had a diagnosis of hypertension, and 389 (9.3%) had a diagnosis of diabetes. Over 30 years of follow-up, there were 813 incident MI events at a median follow-up time of 4725 days.

**Table 1:** Baseline Characteristics of the study participants.

Characteristics	Values (N=4190)
Age (years), mean (SD)	72.6 (5.6)
Gender (male), n (%)	1668 (39.8)
Tobacco consumption, n (%)	2201 (53)
Hypertension, n (%)	2300 (55)
Diabetes, n (%)	389 (9.3)
Total Cholesterol (mg/dL), mean (SD)	211 (38)
BMI, mean (SD)	26.4 (1.9)

### Comparison of Prediction Models Across Time Horizons

**Figure 2:**
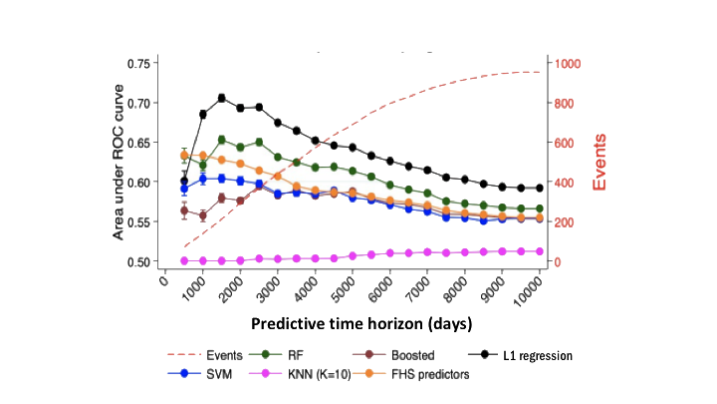
Predictive accuracy over varying time horizons. FHS: Framingham Heart Study; KNN: k-nearest neighbor; RF: random forest; ROC: receiver operating characteristics; SVM: support vector machine.

Relative performance of the machine learning methods and FHS model is displayed in Figure 2 as the AUROC across cut points for the time horizon. The machine learning models were generally most predictive over moderate time horizons of 1500-2500 days of follow-up.

**Figure 3:**
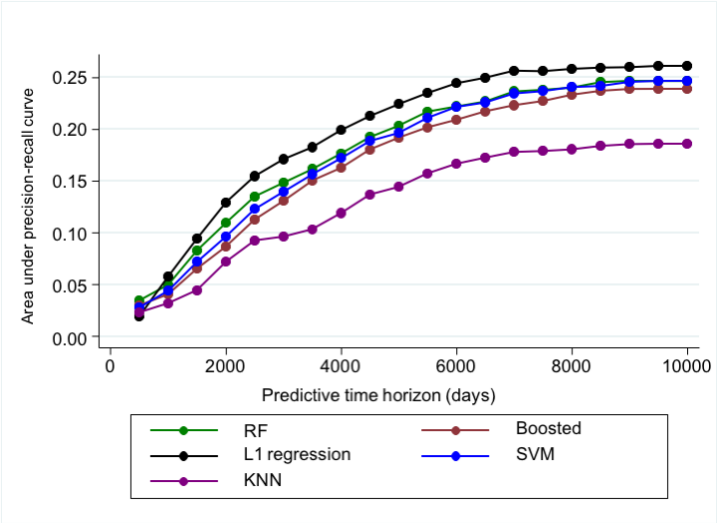
Predictive Accuracy using area under precision-recall curve. KNN: k-nearest neighbor; PR: precision-recall; RF: random forest; SVM: support vector machine.

In addition to examining AUROC, we also examined the area under the precision-recall curve (Figure 3), which favored later time horizons, but with no change in the order of model performance. The L1 regression model still had the highest performance across time points.

**Figure 4:**
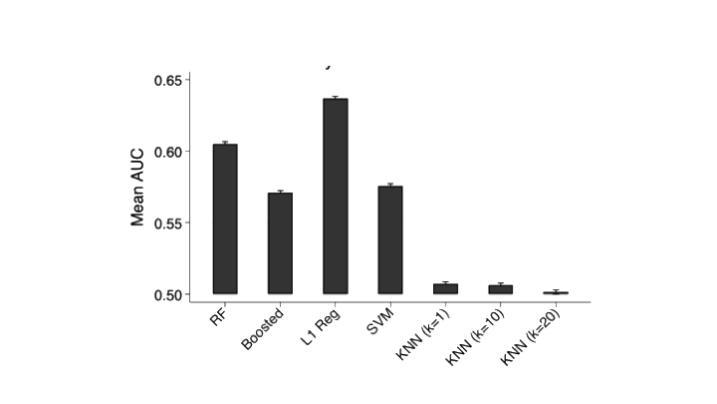
Prediction accuracy across all time horizons. AUC: area under the curve; KNN: k-nearest neighbor; RF: random forest; SVM: support vector machine.

The L1 logistic regression was overall the most predictive across all time points (Figure 4) and displayed the highest prediction accuracy at 1500-day time horizon with an AUROC of 0.71. The k-nearest neighbor model performed relatively poorly across all time points.

When compared with the FHS model, the L1 model performed worse at 500 days of follow-up but had superior prediction accuracy at all subsequent follow-up times. The random forest model performed better than the FHS model starting at 1500 days of follow-up and longer. The remaining machine learning models were less predictive than the FHS model across all time frames (Figure 2).

### Feature Selection

Some machine learning algorithms allow for analysis of variable contributions to the model. For this analysis, feature importance was performed across all time points for the L1 regression and random forest models (Table 2).

**Table 2:** Feature selection (top features).

Model	Short-term follow-up (500-1000 days)	Intermediate follow-up (1500-2500 days)	Long-term follow-up (>2500 days)
L1 regression	Gender (0.90) Calcium channel blockers (0.47) IVCD^a^ by ECG^b^ (0.40) Diabetes mellitus (0.32) Smoking (0.22) Systolic blood pressure (0.21)	Gender (1.03) Diabetes mellitus (0.33) Calcium channel blockers (0.42) Hypertension (0.27) Alcohol (per week) (–0.21)	Gender (0.50) Calcium channel blockers (0.33) Diabetes mellitus (0.20)
Random forest	Weight FEV1^c^ BMI Height LDL-C^d^	Weight FEV1 BMI Height Gender	Weight Total cholesterol BMI Height LDL-C

^a^IVCD: intraventricular conduction delay.

^b^ECG: electrocardiogram.

^c^FEV1: forced expiratory volume in one second.

^d^LDL-C: low-density lipoprotein cholesterol.

For the L1 regression, the most important variables (based on the absolute value of coefficients applied to normalized inputs) at short-term follow-up intervals (ie, <1000 days) were gender, history of diabetes, use of calcium channel blockers or β-blockers, and having a ventricular conduction defect by electrocardiogram. At intermediate follow-up interval (ie, 1500-2500 days), the most important variables were gender, use of calcium-channel blocker, history of diabetes, and history of hypertension. At longer follow-up times (ie, >2500 days), the most important variables were gender, use of calcium channel blocker, and history of diabetes.

For the random forest variable selection based on accuracy, the most important variables at short-term follow-up intervals (ie, <1000 days) were weight, forced expiratory volume (FEV) by pulmonary function testing, BMI, height, and low-density lipoprotein (LDL) cholesterol. At intermediate follow-up interval (1500-2500 days), the most important variables were weight, FEV, BMI, height, and gender. At longer follow-up times (ie, >2500 days), the most important variables were weight, height, BMI, LDL cholesterol, and total cholesterol.

## Discussion

### Principal Findings

This study demonstrates the ability to use machine learning methods for the prediction of incident MI over varying time horizons in cohort data. Using AUROC as the primary metric for model performance, prediction across all models was most accurate in the moderate (ie, 1500-2500 day) follow-up horizon. The L1 regularized regression provided the most accurate prediction across all time frames, followed by the random forest algorithms. These two models compared favorably to the FHS coronary heart disease prediction variables, especially at longer follow-up intervals. Applying ranked variable importance algorithms demonstrated how the variables selected differed over time and in different models.

Prediction was most accurate in the moderate follow-up horizon. We suspect that this was due to the balance of accumulating enough events while still being close in time to the baseline data collected. A predictor that is measured closer in time to the outcome is more likely to be relevant in prediction, and as more events accumulate over time, the power to identify a predictive model increases. Prior studies have looked at machine learning prediction of coronary heart disease at short and intermediate follow-up times; however, to our knowledge, this is the first study to apply models to annual time horizons from short- to long-term follow-up [27].

The L1 regularized regression generally provided the most accurate prediction across all time frames. These regularized regression models expand upon traditional regression models by searching across all variables for the best subset of predictors prior to fitting a regression model. An L1 (Lasso) regression differs from other regularized regression models in that it can shrink the importance of many variables to zero, allowing for feature selection in addition to preventing overfitting. As such, it is very useful when using many variables, like in a cohort or electronic health record data. Prior studies have found these models to be comparable to more advanced machine learning methods for predicting clinical outcomes [28]. The random forest model also performed quite well. Random forest is a regularized form of classification and regression tree model that searches for the covariates that best split the data based on outcome, and then continues to split using additional covariates until many decision “trees” are formed. These models avoid overfitting and can also overcome nonlinearity and handle many variables. The accuracy of the L1 regression and random forest prediction models based on AUROC is reasonable in our study in comparison to prior work [29]. It is worthy of note that we did not include interaction or polynomial terms in the L1 regression, and as such, this model would not be able to identify nonlinear effects between predictors in the same manner as random forest. Our finding that L1 regression provided superior predictive accuracy despite this limitation suggests that nonlinear effects may be less important with these predictors for coronary artery disease or MI, although further work would be needed to support this claim.

With machine learning models, the relationship between any one variable and the outcome is not as clear as with standard regression models. However, some methods can provide the relative importance of each variable to the model creation. We performed ranked variable analysis for the L1 regression and random forest models. We found that, generally, the models found traditional risk factors to be the most important; however, these most important variables changed over time.

The random forest variable importance found weight, height, LDL-cholesterol, and BMI to be highly important across time frames. FEV was important in short- and medium-term follow-up but less important in longer-term follow-up. For the L1 regression, gender, history of diabetes, and the use of calcium channel blockers were important variables across all time horizons. Although these associations are interesting, causation cannot be applied to these analyses, and it can only suggest further study on the importance of these variables.

### Limitations

This study has some notable limitations. First, the CHS [25] data for incident MI are failure time data, and our model does not allow for censored observations due to lack of follow-up. Second, both testing and validation were performed only within the CHS cohort. Although on the one hand, this is an important examination of a specific population, it limits the applicability of our findings to the global population. Machine learning models are very sensitive to the training population and have been found to be biased when created in one population and applied in another. Since the CHS cohort is composed of individuals over the age of 65 years, this analysis provides an opportunity to study machine learning models in this group. We used the original cohort of 5201 participants enrolled in the CHS, which leaves out a subsequent, predominantly African American cohort, making the results less applicable to the global population. Given these limitations, this analysis needs to be validated in novel cohorts. Additionally, this model cannot easily be directly applied to clinical practice; however, this study presents a model for performing similar analysis in more clinically applicable data sets, including electronic health record data. We aim to accomplish this with future studies.

### Conclusions

In a population free of coronary heart disease, machine learning techniques can be used to accurately predict development of incident MI at varying time horizons. Moderate follow-up time horizons appear to have the most accurate prediction given the balance between proximity to baseline data and allowing ample number of events to occur. Future studies are needed to validate this technique in additional populations.

## Appendix

Multimedia Appendix 1 Tables and code used for model analysis.

## Abbreviations

AUROC: area under the receiver operating characteristic curve
CHS: Cardiovascular Health Study
CVD: cardiovascular disease
FEV: forced expiratory volume
FHS: Framingham Heart Study
MI: myocardial infarction
