# Supplementary material for: The Impact of Time Horizon on Classification Accuracy: Application of Machine Learning to Prediction of Incident Coronary Heart Disease
Source: JMIR Cardio. 2022 Nov 2;6(2):e38040. doi: 10.2196/38040 (PMC9669890; doi:10.2196/38040)
Supplement: Multimedia Appendix 1 [file cardio_v6i2e38040_app1.docx]

**Multimedia Appendix 1. Tables and code used for model analysis.**

**Table S1. List of Baseline Variables Used for Prediction**

| **Demographic Variables** | Age (years) | Gender (categorical) | Marital Status (categorical) | Race (categorical) |  |  |
| --- | --- | --- | --- | --- | --- | --- |
| **Exam Variables** | Height (cm) | BMI (Kg/m2) | Waist circumference (cm) | Weight (lbs) | FEV1 (% predicted) | FEV1/FVC (% predicted) |
|  | SBP (mm Hg) | DBP (mm Hg) | Grip Strength | Walk Time (seconds / 15 feet) | FVC (% predicted) |  |
| **Lab Variables** | HDL- Cholesterol (mg/dL) | Glucose | Albumin (g/dL) | CRP (mg/L) | Uric Acid (mg/dL) | Creatinine (mg/dL) |
|  | LDL-Cholesterol (mg/dL) | Triglycerides (mg/dL) | Total Cholesterol (mg/dL) |  |  |  |
| **Social Hx Variables** | Alcohol (#beverages/wk) | Wine (#glasses/wk) | Current Smoker | Ever Smoker | Pack Years smoked at baseline |  |
| **Medication Variables (Taking at baseline)** | ACE-I | Beta Blk Meds | CCB Blk Meds | Diuretic Meds | Aspirin | HTN Meds |
|  | Vasodilator Meds |  |  |  |  |  |
| **Family History Variable** | Stroke | Myocardial Infarction |  |  |  |  |
| **Medical Hx Variables** | Hypertension | Aortic Stenosis | Diabetes | Metabolic Syndrome | Stroke, TIA, or Carotid Endarterectomy |  |
|  | Stroke | Congestive Heart Failure |  |  |  |  |
| **ECG Variables** | Atrial Fibrillation | LVH | Incomplete Right Bundle Branch Block | Q wave MI | Q wave and ST-T wave | ST-T wave abnormality |
|  | ST elevation | 1^st^ degree AV Block | Conduction Defect |  |  |  |
| **Echo Variables** | LV wall motion | LVEF | Mitral Regurgitation |  |  |  |

**Table S2 –Framingham Cox Regression Coefficients for CHD Events**

| Risk Factor | Men | Women |
| --- | --- | --- |
| Age, y | 0.05 | 0.17 |
| Optimal BP (S<120, D<80) | 0.09 | −0.74 |
| Normal BP (S<130, D<85) | 0.00 | 0.00 |
| High normal BP (S<140, D<90) | 0.42 | −0.37 |
| Stage I Htn (S<160, D<100) | 0.66 | 0.22 |
| Stage II-IV Htn (S>160, D>100) | 0.90 | 0.61 |
| Total cholesterol (TC) <160 | −0.38 | −0.21 |
| TC 160-199 | 0.00 | 0.00 |
| TC 200-239 | 0.57 | 0.44 |
| TC 240-279 | 0.74 | 0.56 |
| TC > 280 | 0.83 | 0.89 |
| HDL-C_35 | 0.61 | _ 0.73 |
| HDL-C 35-44 | 0.37 | 0.60 |
| HDL-C 45-49 | 0.00 | 0.60 |
| HDL-C 50-59 | _ 0.00 | 0.00 |
| _HDL-C> 60 | −0.46 | _ −0.54 |
| Diabetes | 0.53 | 0.87 |
| Current smoking | 0.73 | 0.98 |

**Supplemental Data – R Code**

# ML Analysis of CHS data

# Clear memory and graphs

rm(list=ls())

graphics.off()

library(caret)

library(randomForest)

library(RANN)

library(Matrix)

library(glmnet)

library(naniar)

library(plyr)

library (ROCR)

library(e1071)

library(class)

#install.packages("gbm")

library(gbm)

# Load datasets

setwd("~/Science_files/Ongoing_active_projects/Steve_Simon_ML_cohorts/CHS_data/New_code_from_Steve_21JAN2021/")

data1 <- read.csv("./Data_clean_SS_5201.csv", as.is = TRUE)

events <- read.csv("./CHS_Events_5201.csv", as.is = TRUE)

# Create directory for output

currDir <- getwd()

saveFileRoot <- paste0(currDir, "/Analysis_", Sys.Date(), "/")

dir.create(saveFileRoot, showWarnings=TRUE, recursive=FALSE, mode="0777")

# Remove missing CHD

events$incmi[events$incmi == ""] <- NA

events <- events[!is.na(events$incmi),]

####Summary dataset for table 1###

eventscomb <- merge(data1, events, by="idno")

#eventscomb <- eventscomb[(eventscomb$MIHx == "No" & eventscomb$CHDHx == "No"),]

# Remove those with baseline MI =1

eventscomb$anblmod[eventscomb$anblmod == "1"] <- NA

eventscomb <- eventscomb[!is.na(eventscomb$anblmod),]

# Baseline stats

mean(eventscomb$agebl, na.exclude=TRUE)

sd(eventscomb$agebl)

table(eventscomb$gend01)

table(eventscomb$incmi)

table(eventscomb$diabetbl)

table(eventscomb$smoke_y2)

table(eventscomb$hyper_y2)

mean(eventscomb$bmi_y2)

#median(eventscomb$Inc_years, na.exclude=TRUE)

#IQR(eventscomb$Inc_years)

#min(eventscomb$Inc_years)

#max(eventscomb$Inc_years)

#eventscomb$CHDwithin10years <- ifelse((eventscomb$incmi == "1" & eventscomb$ttomi < 3650 & is.na(eventscomb$incmi)==FALSE), 1, 0)

#table(eventscomb$CHDwithin10years)

#eventscomb$CHDwithin1years <- ifelse((eventscomb$incmi == "1" & eventscomb$ttomi < 365 & is.na(eventscomb$incmi)==FALSE), 1, 0)

#table(eventscomb$CHDwithin1years)

#eventscomb$CHDwithin5years <- ifelse((eventscomb$incmi == "1" & eventscomb$ttomi < 1825 & is.na(eventscomb$incmi)==FALSE), 1, 0)

#table(eventscomb$CHDwithin5years)

# Recode missing (blank) as NA

data2 <- replace_with_na_all(data1, condition = ~.x == "")

events_formerge <- events[,c("idno", "incmi", "ttomi")]

combined <- merge(data2, events_formerge, by="idno")

# Data cleaning (manual)

# Drop ID, visit, eSelectin (no data), scarPost (none with), history of MI (can drop others by adding to list)

drops <- c("perstat", "clinic")

combined <- combined[ , !(names(combined) %in% drops)]

combined <- as.data.frame(unclass(combined))

# Impute missing using median imputation

# Code from prior for imputation of median and most common

imputeFunction <- function(data) {

varNames <- names(data)

numVars <- vector()

catVars <- vector()

for(var in varNames) {

#print(var)

if(is.numeric(data[[var]])) {

#print("The variable is numeric")

#print(paste("The value being replaced is", median(data[[var]], na.rm = TRUE)))

data[[var]][is.na(data[[var]])] <- median(data[[var]], na.rm = TRUE)

numVars <- c(numVars, var)

}

if(is.factor(data[[var]])) {

#print("The variable is factor")

#print(paste("The value being replaced is", labels(sort(table(data[[var]]),decreasing=TRUE)[1])))

data[[var]][is.na(data[[var]])] <- labels(sort(table(data[[var]]),decreasing=TRUE)[1])

catVars <- c(catVars, var)

}

if(is.character(data[[var]])) {

#print("The variable is string")

#print(paste("The value being replaced is", labels(sort(table(data[[var]]),decreasing=TRUE)[1])))

data[[var]][is.na(data[[var]])] <- labels(sort(table(data[[var]]),decreasing=TRUE)[1])

catVars <- c(catVars, var)

}

}

return(data)

}

# Run function to impute

combined_Imputed <- imputeFunction(combined)

#any_na(combined_Imputed) # Check

# Standardize variables

# One hot encode categorical variables

cats <- c("idno", "race01", "marit01", "NCEPmsbl", "smoke_y2", "hyper_y2", "lvwmsq2_y2", "asten_y2", "lvsf_y2")

cat_data <- combined_Imputed[cats]

cat_string <- transform(cat_data, marit01 = as.character(marit01),

NCEPmsbl = as.character(NCEPmsbl),

smoke_y2 = as.character(smoke_y2),

hyper_y2 = as.character(hyper_y2),

lvwmsq2_y2 = as.character(lvwmsq2_y2),

asten_y2 = as.character(asten_y2),

lvsf_y2 = as.character(lvsf_y2),

race01 = as.character(race01))

dummies <- dummyVars(" ~ .", data = cat_string)

combined_dummy <- data.frame(predict(dummies, newdata = cat_string))

# Scale float variables

nums <- c("agebl", "pkyrsbl", "alcoh_y2", "wine_y2", "domgrp_y2", "avzmdia_y2",

"avzmsys_y2", "stht_y2", "waist_y2", "bmi_y2", "choladj_y2", "trig_y2",

"hdl_y2", "ldladj_y2", "ldladj_y5", "albadj_y2", "uric_y2", "creadj_y2",

"glu_y2", "weight_y2", "walktime_y2", "fvcpct_y2", "fevpct_y2", "ratpct_y2",

"mregurg_y2", "crpbladj_y2")

num_data <- combined_Imputed[nums]

preProcValues <- preProcess(num_data, method = c("center", "scale"))

num_data_scaled <- predict(preProcValues, num_data)

combined_num <- cbind(combined_Imputed["idno"], num_data_scaled)

# Combine with integers

nums_cats <- c(cats, nums)

int_data <- combined_Imputed[, -which(names(combined_Imputed) %in% nums_cats)]

combined_int <- cbind(combined_Imputed["idno"], int_data)

merge1 <- merge(combined_int, combined_dummy, by="idno")

final_data <- merge(merge1, combined_num, by="idno")

###### Output dataframe for results

outputFileName <- paste0(saveFileRoot, "Results.csv")

heads <- c("Seed", "Horizon", "Events", "AUC_RF", "AUC_Boost", "AUC_L1R", "AUC_SVM",

"AUC_KNN1", "AUC_KNN20", "AUC_KNN50")

commas <- (length(heads)) - 1

cat(heads, file=outputFileName, append=TRUE, sep=c(rep(",", commas), "\n"))

######ML Analysis##########

horizonAnalysis <- function(horizon, seed) {

#Create indicator for event within time horizon

final_data$CHDwithin <- ifelse((final_data$incmi == "1" & final_data$ttomi < horizon & is.na(final_data$incmi)==FALSE), 1, 0)

events <- table(final_data$CHDwithin)[[2]]

print(paste("There were", events, "events in", horizon, "days"))

# Split off outcome and ID

final_data <- final_data[ , -which(names(final_data) %in% c("ttomi", "incmi"))]

X <- final_data[, -which(names(final_data) %in% c("idno", "CHDwithin"))]

y <- final_data["CHDwithin"]

# Remove near zero variance variables

nzv <- nearZeroVar(X)

X_filtered <- X[, -nzv]

# Need to add back for classification

class_data <- cbind(X_filtered, y)

##### Run ML Models################

data_ML <- class_data

# Split data

set.seed(seed)

trainIndex <- createDataPartition(data_ML$CHDwithin, p = .8,

list = FALSE,

times = 1)

Train <- data_ML[ trainIndex,]

Test <- data_ML[-trainIndex,]

numVars = ncol (Train)

testmtry = numVars/3

#prepping data, for both RF and Boosting

names(Train) <- make.names(names(Train))

names(Test) <- make.names(names(Test))

adj_Train = Train

adj_Train$CHDwithin = as.factor(adj_Train$CHDwithin)

Test_x = Test[ ,!(colnames(Test) == "CHDwithin")]

#below is code for iterating through the mtry parameter for random forest

#metric <- "Accuracy"

#for rf caret, need to pass in variable as a factor for it to work and do classification

#control <- trainControl(method="repeatedcv", number=10, repeats=3, search="grid")

#tunegrid <- expand.grid(.mtry=c(1:20))

#rf_gridsearch <- train(CHDwithin~., data= adj_Train, method="rf", metric=metric, tuneGrid=tunegrid, trControl=control)

#print(rf_gridsearch)

#plot(rf_gridsearch)

#m = sqrt(p), better performing in cross val for this value

rf.train = randomForest(formula = CHDwithin ~., data = adj_Train, mtry=sqrt(numVars), importance = TRUE)

#m = p/3

#rf.train = randomForest(formula = CHDwithin ~., data = adj_Train, mtry=(numVars/3), importance = TRUE)

rf.test = predict(rf.train, newdata = Test_x, type = "prob")

#mean((rf.test - Test$CHDwithin)^2)

pred.rf <- prediction(rf.test[,2], Test$CHDwithin)

auc.tmp <- performance(pred.rf,"auc");

print(paste("AUC for Random Forest for", horizon, "-year horizon:", as.numeric(auc.tmp@y.values)))

RF_auc <- as.numeric(auc.tmp@y.values)

#Random Forest Variable Importance

RFgraphname <- paste0(saveFileRoot, 'seed_', seed, '_horizon_', horizon, '_RF_var_imp.jpeg')

jpeg(filename=RFgraphname, width = 600, height = 400)

par(mfrow=c(2,1))

importance(rf.train)

varImpPlot(rf.train)

dev.off()

#boosting

#Using caret below to iterate through optial parameters for boosting

#caretGrid <- expand.grid(interaction.depth=c(1, 3, 5), n.trees = c(100,5000),

# n.minobsinnode=10, shrinkage=c(0.01, 0.001))

#metric <- "Accuracy"

#trainControl <- trainControl(method="cv", number=10)

# tuneGrid = caretGrid,

# verbose=FALSE,

#bag.fraction=0.75

#for gbm, need to pass in CHD as a factor variable for it to work

#gbm.caret <- train(CHDwithin ~., data = adj_Train, distribution="bernoulli", method="gbm", tuneGrid = caretGrid,

# trControl=trainControl, train.fraction = 0.5, metric=metric)

#print(gbm.caret)

#using shrinkage parameter default of 0.001 - selected by tuning parameters per above

boost.train = gbm(CHDwithin ~., data = Train, distribution = 'bernoulli', n.trees = 5000, interaction.depth=4)

#use response parameter to get back predictions

boost.test = predict(boost.train, newdata = Test, n.trees = 5000, type = "response")

#mean((boost.test - Test$CHDwithin)^2)

#don't need: this adj_boost.test = boost.test - 1

pred.boost <- prediction(boost.test, Test$CHDwithin)

auc.tmp <- performance(pred.boost,"auc");

print(paste("AUC for Boosting for", horizon, "-year horizon:", as.numeric(auc.tmp@y.values)))

boost_auc <- as.numeric(auc.tmp@y.values)

# Fit L1 regularized regression with grid search and CV for hyperparameters

x <- model.matrix(CHDwithin~., data_ML)[,-1]

y <- data_ML$CHDwithin

lambda_grid = c(0.00001, 0.0001, 0.001, 0.01, 0.1, 1)

lasso.mod <- cv.glmnet(x[trainIndex,], y[trainIndex], alpha=1, family="binomial", lambda = lambda_grid)

L1graphname <- paste0(saveFileRoot, 'seed_', seed, '_horizon_', horizon, 'L1_mod.jpeg')

jpeg(filename=L1graphname, width = 600, height = 400)

par(mfrow=c(1,1))

plot(lasso.mod)

dev.off()

bestLam <- lasso.mod$lambda.min

lasso.pred <- predict(lasso.mod, s=bestLam, newx=x[-trainIndex,], family="binomial")

pred1 <- prediction(lasso.pred, y[-trainIndex])

auc.tmp <- performance(pred1,"auc");

print(paste("AUC for L1 regression for", horizon, "-year horizon:", as.numeric(auc.tmp@y.values)))

L1R_auc <- as.numeric(auc.tmp@y.values)

# Extract coefficients - STS

#print(coef(lasso.mod, s=lasso.mod$lambda.1se))

# Fit SVM with grid search and CV for hyperparameters

svm.mod <- svm(CHDwithin~., data=data_ML, subset = trainIndex, kernel="radial", gamma = 0.01, cost=0.01)

svm.pred <- predict(svm.mod, data_ML[-trainIndex,])

pred2 <- prediction(as.numeric(svm.pred), data_ML$CHDwithin[-trainIndex])

auc.tmp <- performance(pred2,"auc");

print(paste("AUC for SVM for", horizon, "-year horizon:", as.numeric(auc.tmp@y.values)))

#print(varImp(svm.mod))

SVM_auc <- as.numeric(auc.tmp@y.values)

# Fit KNN at several cutpoints

X_train <- Train[ ,!(colnames(Train) == "CHDwithin")]

y_train <- Train$CHDwithin

X_test <- Test[ ,!(colnames(Test) == "CHDwithin")]

y_test <- Test$CHDwithin

knn.pred <- knn(X_train, X_test, y_train, k=1)

pred3 <- prediction(as.numeric(knn.pred), y_test)

auc.tmp <- performance(pred3,"auc");

print(paste("AUC for KNN (K=1) for", horizon, "-year horizon:", as.numeric(auc.tmp@y.values)))

KNN1_auc <- as.numeric(auc.tmp@y.values)

knn.pred <- knn(X_train, X_test, y_train, k=20)

pred3 <- prediction(as.numeric(knn.pred), y_test)

auc2.tmp <- performance(pred3,"auc");

print(paste("AUC for KNN (K=20) for", horizon, "-year horizon:", as.numeric(auc2.tmp@y.values)))

KNN20_auc <- as.numeric(auc2.tmp@y.values)

# Output to file

summaryData <- c(seed, horizon, events, RF_auc, boost_auc, L1R_auc, SVM_auc,

KNN1_auc, KNN20_auc)

cat(summaryData, file=outputFileName, append=TRUE, sep=c(rep(",", commas), "\n"))

} # End horizon function

for (s in seq(1,100)) {

lapply(c(365,730,1095, 1460, 1825, 3650, 7300, 10220), horizonAnalysis, seed=s)

}

#2190, 2555, 2920, 3285, 3650, 4015, 4380, 4745,5110,5475, 5840,6205,6570,6935,7300,7665,8030,8395,8760,9125,9490,9855,10220,10585,10950
